# Temperature Changes of Pulp Chamber during *In Vitro* Laser Welding of Orthodontic Attachments

**DOI:** 10.1155/2014/589461

**Published:** 2014-01-14

**Authors:** Eren İşman, Rıdvan Okşayan, Oral Sökücü, Serdar Üşümez

**Affiliations:** ^1^Department of Orthodontics, Faculty of Dentistry, Gaziantep University, 27310 Gaziantep, Turkey; ^2^Department of Orthodontics, Faculty of Dentistry, Bezmialem Vakif University, 34093 İstanbul, Turkey

## Abstract

The use of lasers has been suggested for orthodontists to fabricate or repair orthodontic appliances by welding metals directly in the mouth. This work aimed to evaluate the temperature changes in the pulp chamber during welding of an orthodontic wire to an orthodontic molar band using Nd : YAG laser *in vitro*. A freshly extracted human third molar with eliminated pulpal tissues was used. J-type thermocouple wire was positioned in the pulp chamber. A conductor gel was used in the transferring of outside temperature changes to the thermocouple wire. An orthodontic band was applied to the molar tooth and bonded using light cured orthodontic cement. Twenty five mm length of 0.6 mm diameter orthodontic stainless steel wires was welded to the orthodontic band using Nd : YAG laser operated at 9.4 watt. Temperature variation was determined as the change from baseline temperature to the highest temperature was recorded during welding. The recorded temperature changes were between 1.8 and 6.8°C (mean: 3.3 ± 1.1°C). The reported critical 5.5°C level was exceeded in only one sample. The results of this study suggest that intraoral use of lasers holds great potential for the future of orthodontics and does not present a thermal risk. Further studies with larger samples and structural analysis are required.

## 1. Introduction

Numerous studies have been conducted on the use of laser beams for welding dental metals, especially titanium alloys [1–4]. Following Maiman's introduction of the first appliance in 1960, many fields have focused on this technology including industry, the military, communications, and general/dental medicine [5]. Because of their effects on reducing the *heat affected zone* (HAZ) around the welding spot and their ability to maintain material properties, Nd : YAG lasers have been used in metal welding from the beginning. Another advantage of this laser is that it can join different metals much more successfully than other common techniques can [6].

Laser welding techniques began being used in dental offices and laboratories after 1970 [7]. Today, with the help of laser welding, a wide variety of procedures may be performed. For example, dental prostheses and bridges can be constructed or fixed [8, 9], implant titanium milled fixed dentures can be repaired [10], and custom-made laser-welded titanium implant prosthetic abutments can be generated [11]. Additionally, immediate replacement of removable partial dentures can be performed [12], orthodontic space maintainers can be fabricated [13], orthodontic wires can be joined [14], and broken appliances can be fixed [2].

Laser-welding techniques are being used intraorally with an optical fiber transmission. These techniques have been used in many dental offices for various procedures in recent years. In this way, clinicians using this technique can carry out an immediate repair of metallic fixed, removable, and orthodontic broken prostheses and appliances in their own offices, thus reducing the time needed for such repairs [2, 15]. One of the side effects of the welding procedure, however, is the heat produced during the procedure. This heat may cause thermal damage of the periodontal tissues and the tooth pulp. Fornaini et al. tested the thermal changes surrounding the teeth using two hemispherical metal plates placed on to mandibular molars which were laser welded at three points. The temperature changes in the pulp chamber, sulcus, root, and alveolar bone were measured with four K-thermocouple systems [16]. The authors reported that the highest thermal increase was 1.5°C in the pulp chamber, 0.7°C in the sulcus, 0.3°C in the root, and 0.3°C in the alveolar bone. This study demonstrated the biological compatibility of the intraoral laser welding method with the surrounding hard and soft tissues as the parameters used in this work [16].

Bock et al. [13] demonstrated the laser-welding procedure of orthodontic stainless steel wire to orthodontic molar bands for constructing space maintainers. Fornaini declared that the fabrication of space maintainers can be carried out intraorally. He also tested the pulpal and surrounding tissue-related temperature changes during laser welding of metal plates on calf teeth and found no critical pulpal temperature change. However, orthodontic molar bands covered and surrounded the molar tooth and thus we hypothesized that the temperature changes during laser welding may be higher than those observed with Fornaini's method. Moreover, using extracted human teeth may affect the outcome of the experiment since bovine teeth anatomy and structure may not be representative of the thermal conductivity of human teeth, which have been used in many studies [17–23]. Therefore, the aim of this study was to evaluate the temperature changes in the pulp chamber during welding of an orthodontic wire to an orthodontic molar band using an Nd : YAG laser *in vitro*. For the purposes of this study, the null hypothesis assumed that laser welding of orthodontic wire does not cause temperature increases above the critical threshold value of 5.5°C as reported by Zach and Cohen [24].

## 2. Materials and Methods

**Figure 1:**
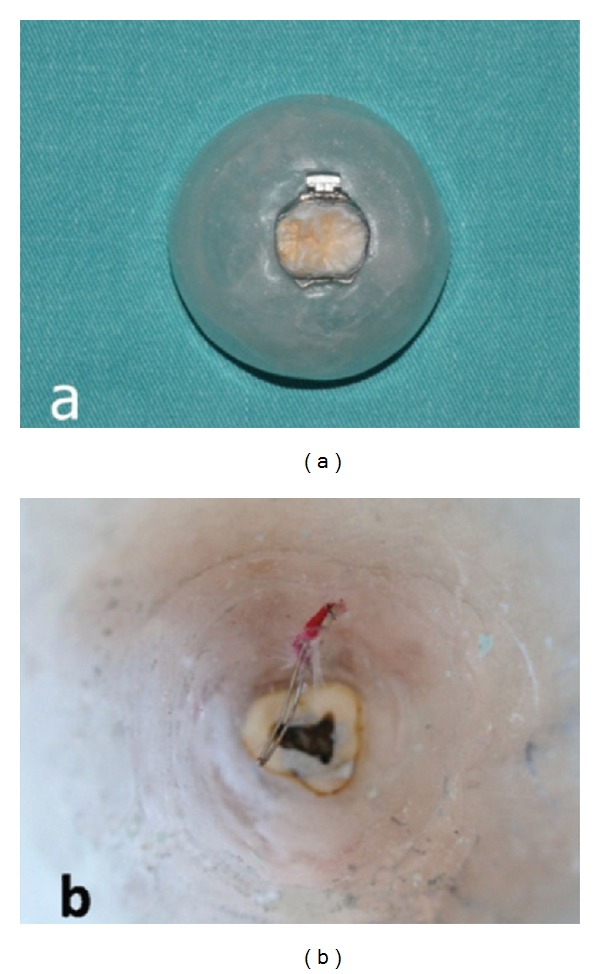
(a) The top view of the acrylic platform and the molar tooth with orthodontic band placed in the middle. (b) The bottom image of the acrylic platform. Note that the inside of the platform was emptied and the crown of the molar tooth was inserted on top of it. The root portion was removed and the cap of the pulp can be seen with conductor gel injected into the cavity providing the transfer of outside temperature changes to the thermocouple wire imitating the pulpal tissue conductibility. The cable was inserted through a hole on the platform.

**Figure 2:**
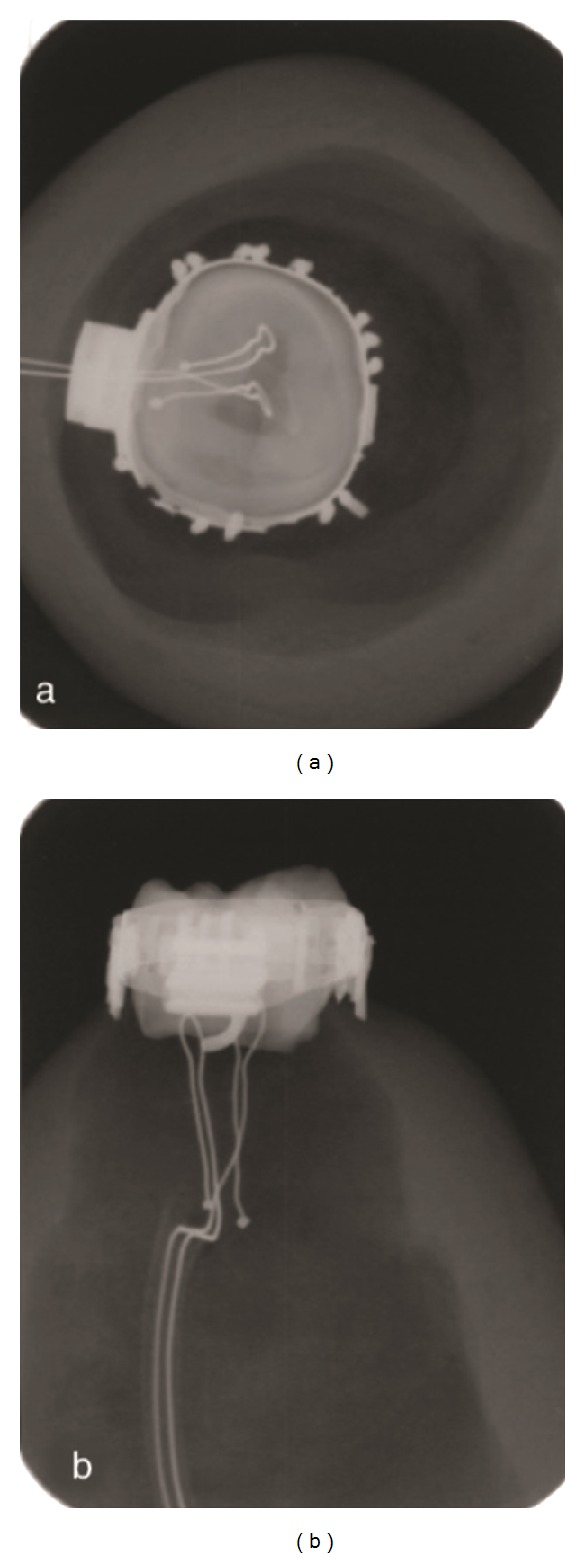
Lateral and occlusal radiograms were taken to ensure that the wire was properly inserted in its place. Two wires in the thermocouple cable can be seen; one of them was placed in the medial fissure of the pulp cap and the other was located in distal fissure.

A freshly extracted human third molar tooth was used in this study. The total thickness of the tooth, including dentine and enamel at the level of the crown, was 1.72 mm on the mesial side, 1.86 mm on the distal side, 1.70 mm on the lingual side, and 2.33 mm on the buccal side. The root was separated from the crown and the intrapulpal tissues were eliminated. An acrylic platform was constructed and emptied inside. The tooth was inserted on top of this platform (Figures 1(a) and 1(b)). In the middle of the platform, a hole was drilled and a J-type thermocouple wire with a 0.36 inch diameter (Omega Engineering, Stamford, CT, USA) was pushed through the hole and positioned in the center of the pulp chamber. There were two wires in the thermocouple cable and one of them was placed in the medial fissure of the pulp roof and the other was located in the distal fissure (Figure 2). A conductor gel (Manhattan Computer Products, China) that imitated the pulpal tissue conductibility was injected into the pulp to ensure transfer of pulpal wall temperature changes to the thermocouple wire. An orthodontic molar band (3M Unitek, Monrovia, CA, USA) was applied to the molar tooth and bonded using multicure glass ionomer orthodontic band cement (3M Unitek, Monrovia, CA, USA). The film thickness of the glass ionomer cement between the crown and orthodontic band was approximately 25–35 *μ*m in accordance with the literature [25]. The space in the acrylic platform was then filled with C-silicone impression material (Zhermack Zetaplus, Rovigo, Italy) to fix the cable in its position. After this, both lateral and occlusal radiograms (Vario DG, Sirona, Bensheim, Germany) were taken to ensure that the wire was properly stabilized in its place (Figure 2).

**Figure 3:**
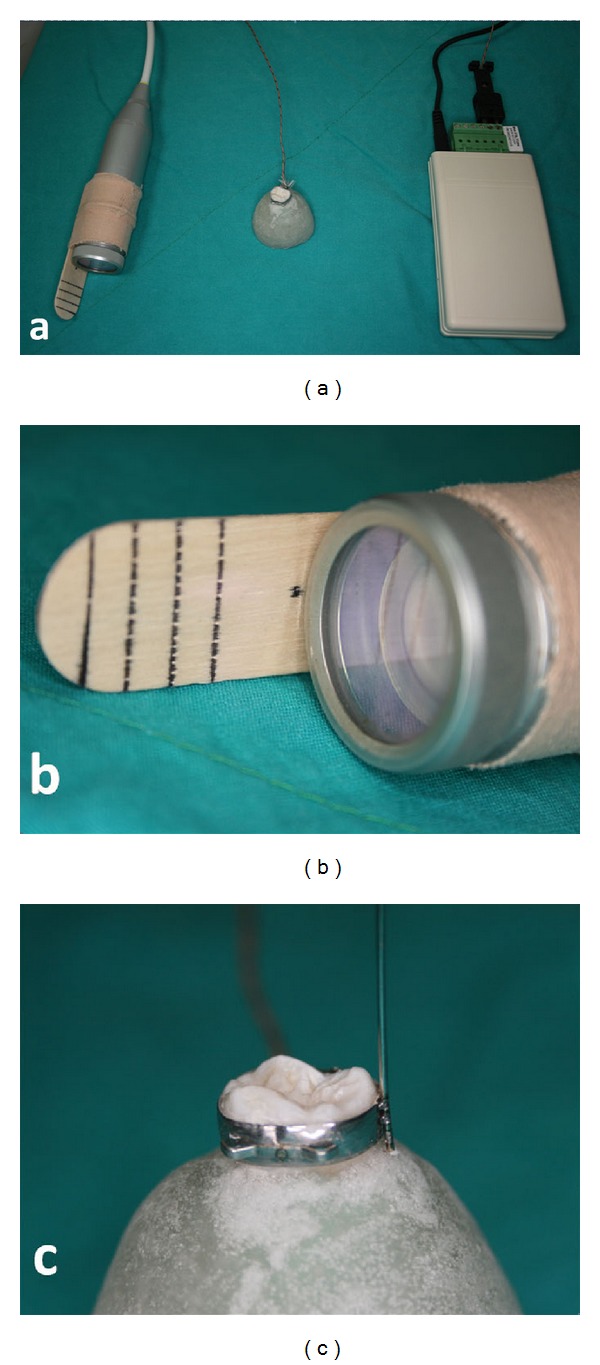
(a) The study design of the recent study. A thermocouple device on the right, the banded tooth with an acrylic platform in the middle, and the laser welding probe. (b) Fiber-delivered probe Nd : YAG laser on which 31 mm stick was attached for standardization. (c) The welded stainless steel 0.016′′ orthodontic wire to the molar band.

Twenty pieces of 0.6 mm diameter orthodontic stainless steel wire (Levanit, Lewadental, Remchingen, Germany) were cut at 5 cm lengths and welded to the orthodontic band using the Nd : YAG laser (Fidelis Plus III, Fotona, Ljubljana, Slovenia). The study design is shown in Figure 3(a).

**Figure 4:**
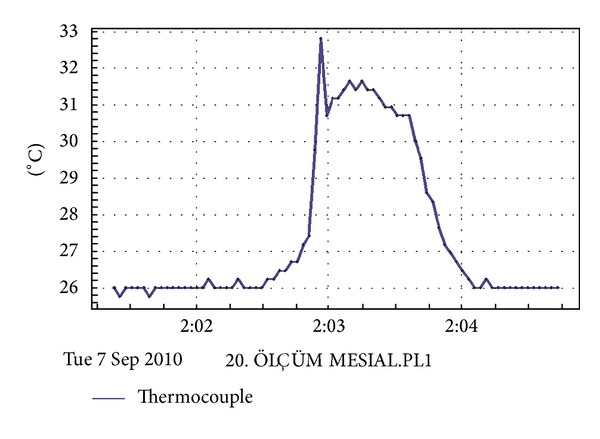
The monitor view of the computer program showing one of the outcomes of the temperature changes during laser welding.

The Nd : YAG laser parameters were as follows: output power: 9.40 W; frequency: 1 Hz; pulse duration: 15 ms; working distance: 31 mm; spot diameter: 0.6 mm; energy: 9.40 J; fluency: 2,300 J/cm^2^ (Figure 4). To standardize the working distance, an accessorial stick measuring 31 mm in length was attached to the laser probe (Figure 3(b)). The metal wire welded to the molar band is shown in Figure 3(c). A small pilot study demonstrated that three pulses are enough to securely weld the piece of wire, and for standardization, the welding process was performed with only three pulses throughout the study. Compressed air was used after each shut to restore the test sample to initial room temperature for standardization.

The J-type thermocouple wire was connected to a data logger (XR440 Pocket Logger, Pace Scientific, NC, USA). During the welding procedures, temperature variations were determined and the changes from the baseline room temperature (26°C) to the highest temperatures were recorded. The sampling rate of the data logger was two seconds. The collected data were monitored in real time and transferred to a computer. The data was available in both tabular and graphic form. Temperature changes were recorded every two seconds from the start of welding and continued for approximately 10–20 seconds until the temperature started to decrease. The outcome data (Figure 4) were analyzed using descriptive statistics including mean, maximum, minimum, and standard deviation.

## 3. Results and Discussion

The outcome was established with a *time-temperature change graph* developed by the computer software and a representative graph is shown in Figure 4. In this graph, the initial temperature of the thermocouple was 26°C and after the application of welding the temperature rapidly increased to a maximum value of 32.80°C. These results were recorded, the alterations were calculated, and statistical analyses were carried out. Mean, maximum, minimum, and standard deviation of the temperature change values are presented in Table 1.

The statistical analyses showed that the temperature changes were between 1.80°C and 6.80°C (mean: 3.33 ± 1.10°C). According to Zach and Cohen's study, the reported critical 5.5°C level [24] was exceeded in only one sample in our study (6.80°C). The rest of the values were below 4.80°C (mostly between 2.23°C and 4.43°C). The null hypothesis was thus accepted.

This work demonstrated the temperature changes occurring in the tooth pulp during laser welding of an orthodontic wire to an orthodontic molar band. This procedure simulates the construction of a banded orthodontic space maintainer in the dental office.

Space maintainers are fixed or removable appliances used to preserve arch length following the premature loss or elective extraction of a tooth or teeth. Fixed appliances are easier to maintain, and they are less likely to be damaged, lost, or removed [26]. Traditionally, the band-loop fixed retainers were generated by adapting a molar band to the related tooth and having a cast model of the patient. The band is then transferred to the cast; a stainless steel orthodontic round wire is organized and bent to maintain the space next to the adjacent tooth and then the metal is attached either by soldering or brazing. This process needs time for construction of the appliance in the laboratory. However, the intraoral laser welding method introduced by Fornaini et al. [2] is more practical and easy to perform. Moreover, it is a time saver for a dental practice.

A study by Nayak et al. demonstrated a method that excluded the impression taking and casting model phase of the band loop space maintainer [27]. However, his method still included the first welding and initial attachment of the band and wire followed by soldering them together firmly and cementing them. This is more practical than the traditional method but a certain amount of chair-side time is still needed.

The current study describes the temperature changes of the pulp cavity during the laser welding of an orthodontic band with an orthodontic stainless steel wire. A similar study was performed by Fornaini et al. [16] using a calf mandible and they showed that the temperature increases in all elements were below the safety level for pulp injury. Our findings are in accordance with Fornaini's study.

There are several advantages of using laser welding technology in dental practices. The use of the same parent metals, either with or without soldering, reduces galvanic corrosion [28]. The small diameter of the HAZ facilitates welding in a very narrow area that may be close to acrylic resin or ceramic parts without damaging the prosthetic structures [29]. It also remarkably reduces chair time or laboratory time, thus expediting the doctors and technicians [15]. However, some precautions must be taken during the welding procedure. It is thought that the probe of the device should be stabilized during laser exposure either by fixing the holding hand to the patient's chin or by supporting it with the opposing hand.

The laser device that was used in this study is capable of combining two different wavelengths which are erbium : yttrium-aluminum-garnet (Er : YAG, 2,940 nm) and Nd : YAG (1,064 nm). Due to insufficient output power for welding metals, diode lasers were left out. It should also be noted that laser welding of titanium using an Nd : YAG laser requires very high pulse energies and an atmosphere of argon gas. Therefore, there are currently limitations to using this technique for welding titanium intraorally.

Fornaini declared that the low temperature rise in the tooth pulp during welding could be explained with the low number of pulses (4 to 5) and the low fluence transferred to the biological structures (fluence = joules per square centimeter; joules = watts per second) [16]. In this study, the fluence was decreased to 2,300 J/cm^2^ compared with Fornaini's fluence parameter of 3,480 J/cm^2^. However, the mean temperature rise of this study (3.33 ± 1.1°C) was higher than Fornaini's (0.71 ± 0.45°C). This might be due to the orthodontic band which tightly covers the crown of the tooth and the closer approximation of the welding spot to the pulp compared to Fornaini's study design. Moreover, our study was conducted using an extracted human tooth instead of bovine or calf teeth which are thought to have thicker dentine and enamel layers.

In this study, the pulpal temperature changes were below the critical value of 5.5°C. However, one of the samples showed a higher change which may be hazardous for pulpal health. It can be assumed that this hazardous change of temperature could be due to malpractice of the clinician who aimed the laser probe directly on the molar band instead of the stainless steel wire. Using the laser in this manner may lead to the creation of a hole on the band and cause unwanted increases in pulpal temperature. As mentioned above, welding applications require the clinician to be extremely attentive while sending the laser beam to the metals intraorally. This may be a disadvantage of the application. However, intraoral laser welding is a very convenient and practical method with all its positive features. Further studies with larger samples and structural analysis are required to settle this procedure down to a large range of dental branches.

## 4. Conclusion

The results of this study suggest that intraoral use of lasers holds great potential for the future of orthodontics and does not present a thermal risk. However, delicate manipulation of the device is advised to minimize discomfort or possible injury to the surrounding tissues in case of extended exposure.

## Figures and Tables

**Table 1:** Descriptive statistics of the outcomes of thermocouple test.

Temperature change (°C)	
Mean	3.33
Max	6.80
Min	1.80
sd	1.10

## References

[1] Sjögren G, Andersson M, Bergman M (1988). Laser welding of titanium in dentistry. *Acta Odontologica Scandinavica*.

[2] Fornaini C, Bertrand C, Bonanini M, Rocca J-P, Nammour S (2009). Welding in the dental office by fiber-delivered laser: a new technique. *Photomedicine and Laser Surgery*.

[3] Bertrand C, le Petitcorps Y, Albingre L, Dupuis V (2004). Optimization of operator and physical parameters for laser welding of dental materials. *British Dental Journal*.

[4] Apotheker H, Nishimura I, Seerattan C (1984). Laser-welded vs soldered nonprecious alloy dental bridges: a comparative study. *Lasers in Surgery and Medicine*.

[5] Bertolotti M (1999). *The History of the Laser*.

[6] Cary HB (2002). *Modern Welding Technology*.

[7] Gordon TE, Smith DL (1970). A laser in dental lab. *Laser Focus Magazine*.

[8] Schneider R (2009). Full mouth restoration on dental implants utilizing titanium laser-welded frameworks. *Journal of Esthetic and Restorative Dentistry*.

[9] Fornaini C, Passaretti F, Villa E (2011). Intraoral laser welding: ultrastructural and mechanical analysis to compare laboratory laser and dental laser. *Lasers in Medical Science*.

[10] Prasad S, Monaco EA (2009). Repairing an implant titanium milled framework using laser welding technology: a clinical report. *Journal of Prosthetic Dentistry*.

[11] Iglesia-Puig MA (2005). Custom-made laser-welded titanium implant prosthetic abutment. *Journal of Prosthetic Dentistry*.

[12] Hassan L, Juszczyk AS, Clark RKF (2005). Immediate replacement removable partial dentures with cobalt-chromium frameworks: rationale, technology and a case report. *Journal of Oral Rehabilitation*.

[13] Bock JJ, Bailly J, Gernhardt CR, Fuhrmann RAW (2008). Fracture strength of different soldered and welded orthodontic joining configurations with and without filling material. *Journal of Applied Oral Science*.

[14] Watanabe E, Stigall G, Elshahawy W, Watanabe I (2011). Deflection load characteristics of laser-welded orthodontic wires. *Angle Orthodontist*.

[15] Fornaini C, Vescovi P, Merigo E (2010). Intraoral metal laser welding: a case report. *Lasers in Medical Science*.

[16] Fornaini C, Bertrand C, Rocca JP (2010). Intra-oral laser welding: an in vitro evaluation of thermal increase. *Lasers in Medical Science*.

[17] Malkoç S, Uysal T, Üşümez S, Işman E, Baysal A (2010). In-vitro assessment of temperature rise in the pulp during orthodontic bonding. *American Journal of Orthodontics and Dentofacial Orthopedics*.

[18] Baysal A, Uysal T, Usumez S (2007). Temperature rise in the pulp chamber during different stripping procedures: an in vitro study. *Angle Orthodontist*.

[19] Yondem I, Altintas SH, Usumez A (2011). Temperature rise during resin composite Polymerization under different ceramic restorations. *European Journal of Dentistry*.

[20] Tosun G, Usumez A, Yondem I, Sener Y (2008). Temperature rise under normal and caries-affected primary tooth dentin disks during polymerization of adhesives and resin-containing dental materials. *Dental Materials Journal*.

[21] Altintas SH, Yondem I, Tak O, Usumez A (2008). Temperature rise during polymerization of three different provisional materials. *Clinical Oral Investigations*.

[22] Eldeniz AU, Usumez A, Usumez S, Ozturk N (2005). Pulpal temperature rise during light-activated bleaching. *Journal of Biomedical Materials Research B*.

[23] Ozturk B, Ozturk AN, Usumez A, Usumez S, Özer F (2004). Temperature rise during adhesive and resin composite polymerization with various light curing sources. *Operative Dentistry*.

[24] Zach L, Cohen G (1965). Pulp response to externally applied heat. *Oral Surgery, Oral Medicine, Oral Pathology*.

[25] Singh G (2007). *Textbook of Orthodontics*.

[26] Laing E, Ashley P, Naini FB, Gill DS (2009). Space maintenance. *International Journal of Paediatric Dentistry*.

[27] Nayak UA, Loius J, Sajeev R, Peter J (2004). Band and loop space maintainer: made easy. *Journal of the Indian Society of Pedodontics and Preventive Dentistry*.

[28] Zupancic R, Legat A, Funduk N (2006). Tensile strength and corrosion resistance of brazed and laser-welded cobalt-chromium alloy joints. *Journal of Prosthetic Dentistry*.

[29] Tambasco J, Anthony T, Sandven O (1996). Laser welding in the dental laboratory: an alternative to soldering. *Journal of Dental Technology*.

